# Effects of Heme Site (FA1) Ligands Bilirubin, Biliverdin, Hemin, and Methyl Orange on the Albumin Binding of Site I Marker Warfarin: Complex Allosteric Interactions

**DOI:** 10.3390/ijms232214007

**Published:** 2022-11-13

**Authors:** Beáta Lemli, Zuzana Lomozová, Tamás Huber, András Lukács, Miklós Poór

**Affiliations:** 1 Department of Pharmacology, Faculty of Pharmacy, University of Pécs, Rókus u. 2, H-7624 Pécs, Hungary; 2 Green Chemistry Research Group, János Szentágothai Research Centre, University of Pécs, Ifjúság útja 20, H-7624 Pécs, Hungary; 3 The Department of Pharmacognosy and Pharmaceutical Botany, Faculty of Pharmacy in Hradec Králové, Charles University, Akademika Heyrovského 1203, 500 05 Hradec Králové, Czech Republic; 4 Department of Biophysics, Medical School, University of Pécs, Szigeti út 12, H-7624 Pécs, Hungary; 5 Lab-on-a-Chip Research Group, János Szentágothai Research Centre, University of Pécs, Ifjúság útja 20, H-7624 Pécs, Hungary

**Keywords:** human serum albumin, warfarin, Sudlow’s site I, heme site, allosteric modulation

## Abstract

Human serum albumin (HSA) is the most abundant plasma protein in circulation. The three most important drug-binding sites on HSA are Sudlow’s Site I (subdomain IIA), Sudlow’s Site II (subdomain IIIA), and Heme site (subdomain IB). Heme site and Site I are allosterically coupled; therefore, their ligands may be able to allosterically modulate the binding affinity of each other. In this study, the effects of four Heme site ligands (bilirubin, biliverdin, hemin, and methyl orange) on the interaction of the Site I ligand warfarin with HSA were tested, employing fluorescence spectroscopic, ultrafiltration, and ultracentrifugation studies. Our major results/conclusions are the following. (1) Quenching studies indicated no relevant interaction, while the other fluorescent model used suggested that each Heme site ligand strongly decreases the albumin binding of warfarin. (2) Ultrafiltration and ultracentrifugation studies demonstrated the complex modulation of warfarin–HSA interaction by the different Heme site markers; for example, bilirubin strongly decreased while methyl orange considerably increased the bound fraction of warfarin. (3) Fluorescence spectroscopic studies showed misleading results in these diligand–albumin interactions. (4) Different Heme site ligands can increase or decrease the albumin binding of warfarin and the outcome can even be concentration dependent (e.g., biliverdin and hemin).

## 1. Introduction

Human serum albumin (HSA) is the most abundant plasma protein (35–50 mg/mL) in circulation [1,2]. HSA is a multifunctional protein, which maintains the oncotic pressure of the blood. It has buffer and antioxidant functions and it binds and transports numerous endogenous and exogenous compounds [1,2,3]. HSA is a single polypeptide chain protein (585 amino acids; 66.5 kDa) and its structure is stabilized by 17 disulfide bridges. HSA is built up from three homologous domains (I, II, and III) and each domain consists of two subdomains (A and B) [1,4]. The hydrophobic cavities of the subdomains IIA and IIIA are the two main and most well-known drug-binding sites of HSA, called Sudlow’s Site I and Sudlow’s Site II, respectively. Numerous drugs, nutrients, and toxins occupy these primary binding sites [2]. In addition, an apolar pocket in subdomain IB, namely the Heme site, has also been reported as an important third drug-binding site on HSA [2,5]. Furthermore, HSA binds and carries fatty acids (FAs) with high-, medium-, and low-affinity FA binding sites, some of these overlapping with the major drug-binding sites listed above [2,6].

**Figure 1:**
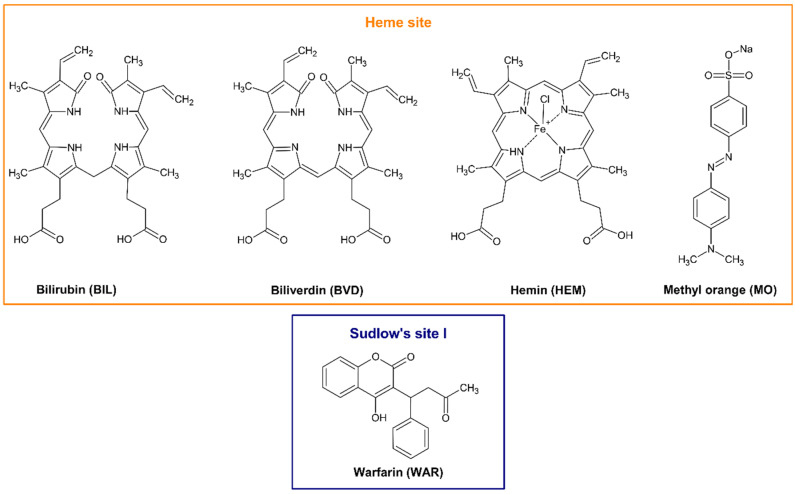
Chemical structures of bilirubin (BIL), biliverdin (BVD), hemin (HEM), methyl orange (MO), and warfarin (WAR).

Site I (FA7) is an apolar pocket in subdomain IIA containing some polar residues. The binding of fatty acids can alter both volume and polarity of this drug-binding site [7,8]. Heterocyclic compounds and organic acids are common ligands of Site I, where the most preferred position of their main planar part is between the sidechains of Leu238 and Ala291, although the relatively large size of the binding pocket offers several other positions [9]. Warfarin (WAR; Figure 1) is a typical ligand of Site I and its two enantiomers bind to the same position in deprotonated state [8]. The WAR–HSA complex is stabilized by hydrophobic interaction with the Trp214 and hydrogen bond with the His242 [8]. Previous spectroscopic, affinity chromatography and ultracentrifugation experiments demonstrated that the binding constant (*K*) of WAR–HSA complex is approximately 2 × 10^5^ L/mol (log*K* ≈ 5.3) [10,11,12,13].

Heme site (or Heme pocket; FA1) is located in subdomain IB. Heme and certain FAs can rearrange the local conformation of subdomain IB with the reorientation of Tyr138 and Tyr161 amino acids, which generates the cavity for these endogenous ligands [9]. Bilirubin (BIL; Figure 1) has similar chemical structure and binding site on HSA to heme; however, it does not induce the above-described changes in the structure of fatty-acid-free HSA [9]. Biliverdin (BVD; Figure 1) and hemin (HEM; Figure 1) occupy the same area of subdomain IB as heme [5,14] and the binding site of the pH indicator methyl orange (MO; Figure 1) has also been identified in subdomain IB [15]. In previous studies, controversial data have been reported in regard to the affinity of certain Heme site ligands toward albumin. The binding constant of the BIL–HSA complex was suggested from 10^6^ to 10^8^ L/mol (log*K* = 6 to 8) [16,17,18,19,20,21,22]. Most of the previously reported studies agree that the binding constant of the BVD–HSA complex is approximately 10^6^ L/mol (log*K* ≈ 6) [20,23,24]. However, when the interaction of HEM with human or bovine serum albumins was examined, the binding constants determined were in the 10^5^ to 10^8^ L/mol range (log*K* = 5 to 8) [5,25,26,27]. Another study suggests 2.3 × 10^5^ L/mol (log*K* = 5.4) as the binding constant of the MO–HSA complex [15].

The conformational adaptability and flexibility of HSA with its multidomain structure and multiple binding sites result in HSA being an allosteric protein [2]. In the presence of two different ligand molecules, the interaction of the ligands with the protein can be cooperative (both compounds are able to interact with HSA at the same time) or competitive (the binding sites of the ligands are the same or overlap and they cannot be bound simultaneously). The competitive interaction is relatively simple: The ligands compete for the same binding site; consequently, their concentrations and affinities will determine the outcome. However, the cooperative binding of ligands can lead to allosteric interactions, where the simultaneous binding of the other ligand influences the affinity of the first ligand toward HSA. The formation of a ligand–albumin complex may induce some conformational changes in other binding site(s), leading to the development of positive or negative allosteric modulation. As previous studies demonstrated, Site I and Heme site are allosterically coupled [2]. Therefore, Heme site ligands may affect the interactions of Site I ligands with HSA and vice versa [28]. Earlier studies suggest that heme can strongly decrease the binding affinity of certain Site I ligands toward HSA, including anti-HIV drugs (e.g., abacavir, efavirenz, and zidovudine), furosemide, indomethacin, and phenylbutazone [29,30]. However, in another report, the presence of heme only slightly reduced the binding constant of the WAR–HSA complex [31]. Since the available data in regard to the effects of Heme site ligands on the albumin binding of Site I ligand drugs are very limited, further extensive studies are required.

Several experimental methodologies are applied for the investigation and characterization of ligand–albumin interactions, including equilibrium dialysis, ultrafiltration, ultracentrifugation, circular dichroism, affinity chromatography, surface plasmon resonance, capillary electrophoresis, isothermal titration calorimetry, differential scanning calorimetry, and X-ray crystallography [32]. Nevertheless, UV-Vis and fluorescence spectroscopy are the most commonly used techniques. The fluorescence signal of HSA is mainly exerted by its single tryptophan residue (Trp214; located in Site I, subdomain IIA) with lower involvement of tyrosine and phenylalanine amino acids [33]. The fluorescence of Trp214 is highly sensitive to microenvironmental changes; therefore, the formation of stable ligand–albumin complexes typically affects the emission signal of albumin [34]. This is the theoretical basis of fluorescence quenching studies, which are very frequently employed to characterize ligand–albumin interactions. Furthermore, the complex formation with HSA can affect the UV-Vis or fluorescence signal of a ligand molecule; therefore, the albumin-induced changes in the absorbance or in the fluorescence emission signal of a ligand can also be applied for the investigation of these interactions [12,35]. UV-Vis and fluorescence spectroscopy are relatively cheap and powerful techniques in regard to the investigation of ligand–albumin interactions; nevertheless, the cooperative binding of two ligands (diligand–albumin systems) can cause unexpected spectral changes. These limitations need to be carefully considered during the evaluation of spectroscopic data.

Sudlow’s Site I (also called “acidic drug binding site”) interacts with several hundreds of drugs (and other xenobiotics), including non-steroidal anti-inflammatory drugs (e.g., phenylbutazone), oral anticoagulants (e.g., WAR), oral antidiabetics (e.g., glimepiride), diuretics (e.g., furosemide), etc. [36]. Changes in the albumin binding of these drugs can strongly affect their pharmacokinetic properties; therefore, the displacement or the increased binding affinity of Site I ligand drugs may have significant pharmacological importance. One typical example is WAR, which has a narrow therapeutic window that is accompanied by its high plasma protein binding (≈99%) [12]. Furthermore, the interactions of Heme site ligands with WAR have been barely characterized yet. Using the most commonly applied spectroscopic techniques, the precise evaluation of allosteric interactions is very challenging. In the present work, we aimed to examine the effects of four Heme site ligands (BIL, BVD, HEM, and MO) on the albumin binding of the Site I marker WAR. First, the binding constants of BIL–HSA, BVD–HSA, HEM–HSA, and MO–HSA complexes were determined based on UV-Vis spectroscopic and fluorescence quenching studies. The fluorescence quenching effect of WAR on HSA was tested in the absence and presence of Heme site ligands, after which the impacts of BIL, BVD, HEM, and MO on the WAR–HSA complex were also examined based on the changes in the fluorescence emission signal of WAR. Thereafter, interactions of Heme site ligands with the WAR–HSA complex were also evaluated, employing ultrafiltration and ultracentrifugation techniques. Heme site markers caused very complex and sometimes opposite regulations on WAR–HSA interaction. In addition, our results underline that fluorescence spectroscopic evaluation of these diligand–albumin systems can be misleading. This study provides a deeper insight into the allosteric interactions of Heme site ligands with the Site I marker WAR.

## 2. Results and Discussion

### 2.1. Interaction of Heme Site Ligands with HSA Based on UV-Vis Spectroscopic and Fluorescence Quenching Studies

**Figure 2:**
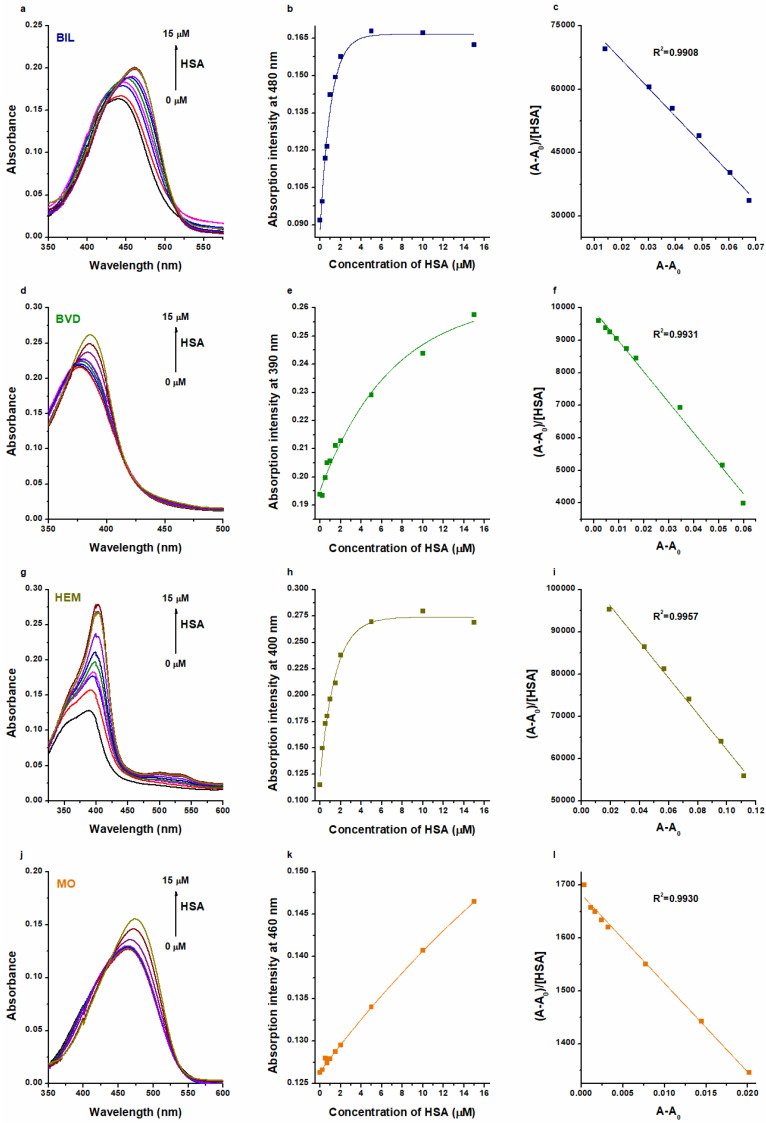
Representative UV-Vis absorption spectra of BIL (**a**), BVD (**d**), HEM (**g**), and MO (**j**) in the presence of HSA in PBS (pH 7.4; Heme site ligands: 5 μM; HSA: 0–15 μM). HSA-induced increase in the absorbances of BIL at 480 nm (**b**), BVD at 390 nm (**e**), HEM at 400 nm (**h**), and MO at 460 nm (**k**). Scatchard plots (Equation (1)) of BIL–HSA (**c**), BVD–HSA (**f**), HEM–HSA (**i**), and MO–HSA (**l**) complexes. Both Scatchard and Hyperquad evaluations were performed at more wavelengths, where we did not notice relevant differences in the log*K* values determined. Therefore, we presented here the wavelengths with the best fitting in regard to the Scatchard plots.

First, we tested the interaction of BIL, BVD, HEM, and MO with HSA, employing UV-Vis and fluorescence spectroscopic studies. In UV-Vis experiments, BIL, BVD, HEM, and MO showed their absorption maxima at 440 nm, 376 nm, 388 nm, and 462 nm, respectively. HSA did not show absorbance at these wavelengths. However, in a concentration-dependent fashion, albumin increased the absorption signal of each Heme site ligand tested and induced a redshift (BIL: 440 → 460 nm; BVD: 376 → 386 nm; HEM: 388 → 403 nm; MO: 462 → 474 nm) in their absorption wavelength maxima (Figure 2). Based on these data, the binding constants were calculated using the Scatchard plot (linear fitting; Equation (1)) and the Hyperquad2006 software (non-linear fitting; Equations (2)–(7)).

**Figure 3:**
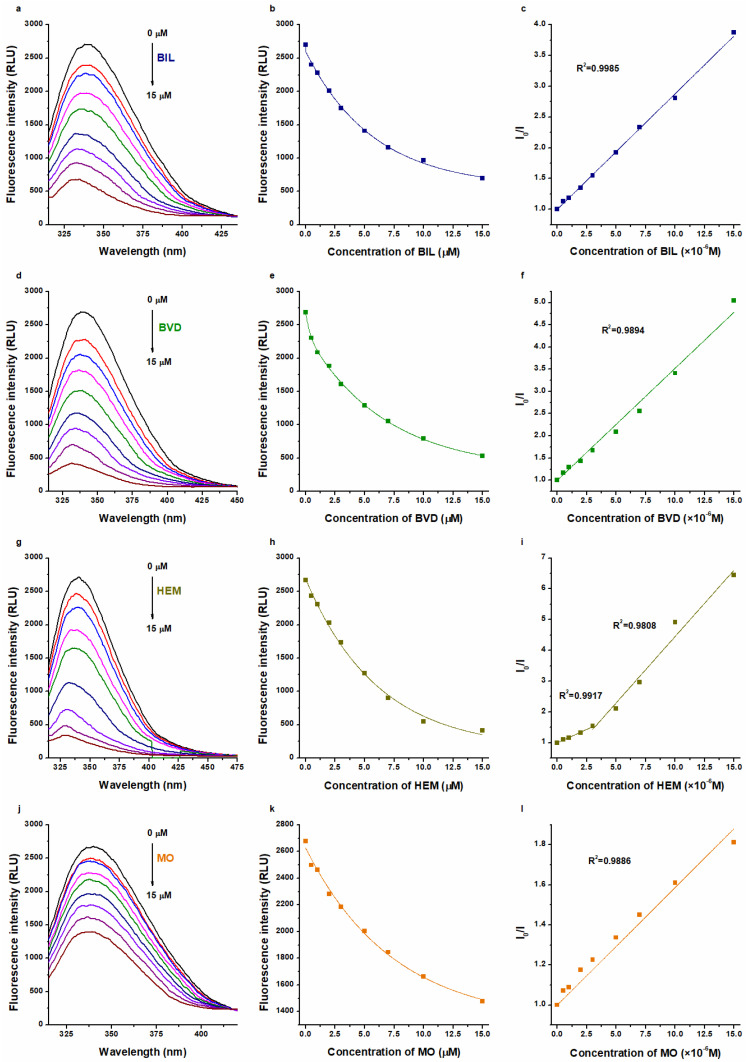
Representative fluorescence emission spectra of HSA (2 μM) in the presence of increasing concentrations (0–15 μM) of BIL (**a**), BVD (**d**), HEM (**g**), and MO (**j**) in PBS (pH 7.4; λ_ex_ = 295 nm). Ligand-induced decrease in the emission signal of HSA at 340 nm: BIL (**b**), BVD (**e**), HEM (**h**), and MO (**k**) exerted strong quenching impacts (the inner-filter effects of Heme site ligands were corrected based on their absorption spectra recorded). Stern–Volmer plots (Equation (9)) of BIL–HSA (**c**), BVD–HSA (**f**), HEM–HSA (**i**), and MO–HSA (**l**) complexes.

The interactions of BIL, BVD, HEM, and MO with HSA were also tested based on their fluorescence quenching impacts on the protein. Even after the correction of inner-filter effects (see in Equation (8)), each Heme site ligand caused a strong, concentration-dependent decrease in the emission signal of HSA at 340 nm (Figure 3). Using these data, binding constants were calculated using the Stern–Volmer plot (linear fitting; Equation (9)) and the Hyperquad software (non-linear fitting; Equations (2)–(7)).

The decimal logarithmic values of *K* and *K_SV_* (determined based on UV-Vis and fluorescence quenching studies) are summarized in Table 1. Typically, Scatchard and Stern–Volmer plots showed good linearity, suggesting a 1:1 stoichiometry of complex formation (Figure 2 and Figure 3). The sole exception was noticed in the quenching studies of HEM, where two linear sections appeared in the Stern–Volmer plot (Figure 3i). Sometimes, the presence of a second linear part indicates a further binding site [37,38,39,40] or it results from the combined dynamic and static quenching processes [41,42,43,44]. We evaluated these sections both together and separately; however, only slight differences were observed in the log*K_SV_* and log*K* values (Table 1). 

Evaluation with the Hyperquad software also suggested the best fitting with the 1:1 stoichiometry model. R^2^ values of fittings were 0.98 or higher, except in the UV-Vis studies of BIL–HSA (R^2^ = 0.63) and HEM–HSA (R^2^ = 0.78). Nevertheless, we cannot exclude the existence of lower-affinity secondary binding sites, as suggested by some studies in regard to BIL and HEM [17,25].

In quenching studies, log*K_SV_* and log*K* values were in agreement for each ligand–albumin complex. Furthermore, for BVD–HSA and MO–HSA complexes, fluorescence quenching and UV-Vis studies showed similar binding constants, where the evaluation with the Scatchard plot gave slightly lower log*K* values (Table 1). These data are also in agreement with the previously reported binding constants of BVD–HSA [20,23,24] and MO–HSA [15] complexes.

In regard to BIL–HSA and HEM–HSA, quenching studies suggested similar log*K* values to the data derived from UV-Vis studies evaluated with the Scatchard plot (Table 1). However, the Hyperquad evaluation of UV-Vis data suggests approximately 100-fold higher binding constants of BIL–HSA (log*K* = 7.5) and HEM–HSA (log*K* = 7.4). These high log*K* values seem to be more reliable because even equimolar concentration of HSA (5 μM) with these ligands induced close to maximal change in the absorbance values of BIL and HEM (Figure 2b,h). Importantly, in quenching studies, we examined the impact of the ligand on the fluorescence signal of the protein. From this point of view, the most important moiety of HSA (Trp214) is located in Site I (subdomain IIA), while BIL and HEM occupy subdomain IB as their high-affinity binding site. On the other hand, in the UV-Vis experiments, we followed the albumin-induced changes in the absorbance of Heme site ligands. Considering the previously reported binding constants for BIL–HSA [16,17,18,19,20,21,22] and HEM–HSA [5,25,26,27] complexes, it is reasonable to hypothesize that we likely examined the interactions of these ligands with a high-affinity and a low-affinity binding site with UV-Vis and fluorescence quenching studies, respectively. This idea is also supported by previous reports, which suggested more binding sites in BIL and HEM on albumin [17,25]. In addition, the above-listed observations strongly underline that different spectroscopic techniques and data evaluation strategies can strongly affect the binding constants determined for ligand–albumin complexes.

### 2.2. Effects of Heme Site Ligands on the WAR-Induced Quenching Effect

**Figure 4:**
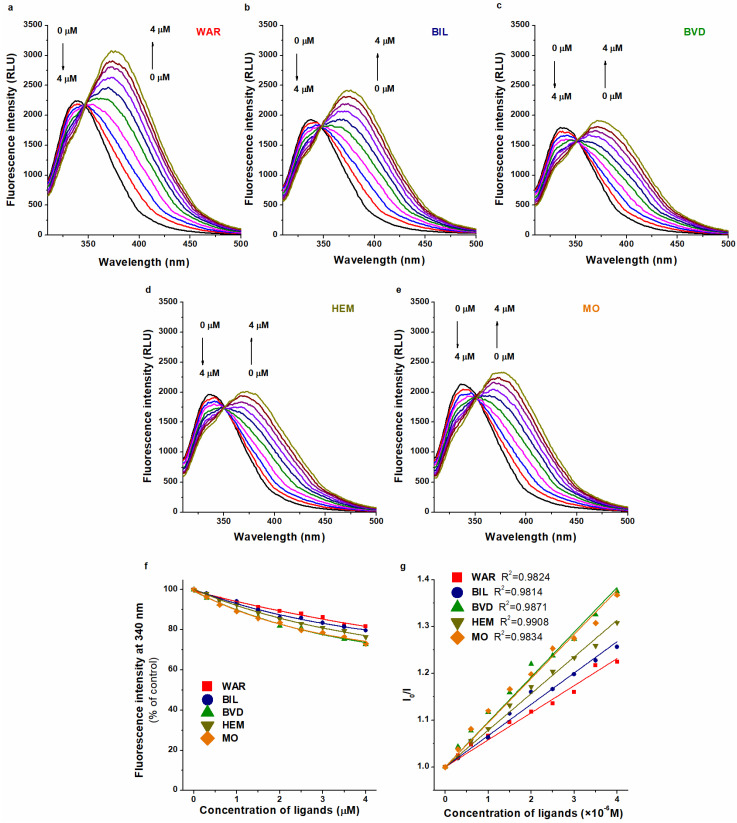
Fluorescence quenching effect of WAR (0.0–4.0 μM) on HSA (2.0 μM) in the absence (**a**) and presence of BIL (**b**), BVD (**c**), HEM (**d**), and MO (**e**) in PBS (pH 7.4; λ_ex_ = 295 nm; concentration of Heme site ligands: 1.0 μM). WAR-induced decrease in the fluorescence emission signal of HSA at 340 nm (% of control) without and with Heme site ligands (**f**). Stern–Volmer plots (Equation (9)) of WAR–HSA in the absence and presence of BIL, BVD, HEM, and MO ((**g**); λ_ex_ = 295 nm; λ_em_ = 340 nm). Arrows on the left side of panels (**a**–**e**) demonstrate the decreasing emission signal of HSA at 340 nm, while the arrows on the right side of the same panels refer to the increasing emission peak of WAR around 380 nm.

In the following experiment, the fluorescence quenching effect of WAR on HSA was tested in the absence and presence of BIL, BVD, HEM, or MO (λ_ex_ = 295 nm). This experimental design is commonly applied to examine the potential interactions when two ligands and HSA are presented [45,46,47,48], then the binding constant of the test ligand is evaluated in the absence and presence of a known site marker. Under these experimental conditions, the emission spectra of HSA and WAR (both bound and unbound forms) overlap (Figure 4): the first emission peak at 340 nm belongs to HSA, while the increasing second peak around 380 nm is produced by WAR. BIL, BVD, HEM, or MO alone did not exert fluorescence at 340 and 380 nm. The emission intensities of WAR alone and in the presence of Heme site ligands (without albumin) at 340 nm are demonstrated in Figure S1. Furthermore, it is important to note that Heme site ligands can strongly affect the emission intensity of the WAR–HSA complex (see later in Section 2.3). Consequently, in this model, both the first (HSA) and the second (WAR) peaks are modified by Heme site markers. Therefore, the precise quantitative evaluation of these data seems to be extremely complicated. Due to the complexity of the system, we could not properly deconvolute the spectra. Nevertheless, in the presence of Heme site markers, the WAR-induced intensity changes at 340 nm did not show large differences (Figure 4f). In addition, Stern–Volmer plots (Figure 4g) also suggest only minor changes in binding affinity of WAR when the Heme site ligands were added (log*K_SV_* values were between 4.8 and 5.0). Thus, these semi-quantitative observations may suggest the minor effects of Heme site markers on the albumin binding of WAR.

### 2.3. Effects of Heme Site Ligands on the Fluorescence of WAR–HSA Complex

In the next step, we tested the impacts of Heme site ligands based on the changes in the emission signal of the WAR–HSA complex. Albumin binding results in a large increase in the emission signal of WAR (λ_ex_ = 317 nm; λ_em_ = 379 nm; Figure S2) [12]. Therefore, the changes in the ratio of unbound and albumin-bound forms can markedly affect the emission signal of WAR [12,49]. Increasing concentrations of BIL, BVD, HEM, or MO were added to the WAR–HSA complex in PBS (pH 7.4). The emission intensities of WAR alone and in the presence of Heme site ligands (without albumin) at 379 nm are demonstrated in Figure S2. For comparison, the same experiments were also performed with two Sudlow’s Site II ligands (diazepam and ketoprofen) and with two fatty acids (palmitic acid and stearic acid). 

**Figure 5:**
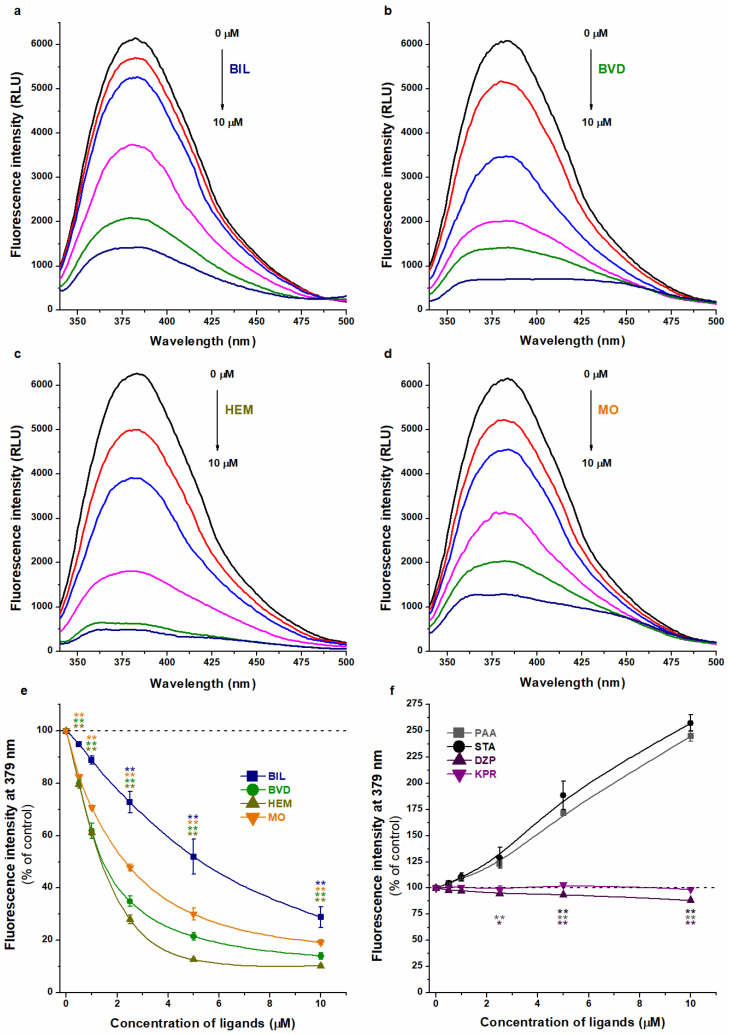
Representative fluorescence emission spectra of WAR (1 μM) in the presence of HSA (3.5 μM) and increasing concentrations (0–10 μM) of BIL (**a**), BVD (**b**), HEM (**c**), and MO (**d**) in PBS (pH 7.4; λ_ex_ = 317 nm). Effects of Heme site ligands ((**e**); BIL, BVD, HEM, and MO), Sudlow’s site II ligands (**f**; DZP, diazepam; KPR, ketoprofen), and fatty acids ((**f**); PAA, palmitic acid; STA, stearic acid) on the fluorescence emission signal of WAR–HSA complex in PBS (λ_ex_ = 317 nm, λ_em_ = 379 nm; * *p* < 0.05, ** *p* < 0.01).

Each Heme site ligand tested strongly reduced the emission signal of WAR (Figure 5), suggesting that BIL, BVD, HEM, and MO may decrease the bound fraction of the Site I ligand. BIL induced the lowest impact, followed by MO, BVD, then HEM. Quenching studies suggested only minor effects of Heme site markers (Figure 4), while changes in the fluorescence of WAR indicated its considerable displacement from the protein (Figure 5). However, before we make any conclusions, we have to see the results of the further experiments.

Site II ligands did not change (ketoprofen) or slightly decreased (diazepam) the fluorescence of WAR (Figure 5f), which refers to their negligible effect on the albumin binding of the Site I marker. 

Both fatty acids (palmitic acid and stearic acid) induced a very large (almost 2.5-fold) increase in the emission intensity at 379 nm (Figure 5f). Under these conditions, most of the WAR molecules are albumin bound; therefore, this marked elevation could not be simply caused by a potential increase in the albumin-bound fraction of WAR. Furthermore, palmitic acid and stearic acid did not show background fluorescence at 379 nm, neither alone nor in the presence of HSA (Figure S3). These observations highlight that the cooperative binding of fatty acids and WAR can strongly increase the fluorescence signal of the WAR–HSA complex. Since allosteric interactions may influence the stability and/or the fluorescence properties of a ligand–albumin complex, the interpretation of spectral changes should be carefully considered to avoid false conclusions.

### 2.4. Effects of Heme Site Ligands on WAR–HSA Interaction Based on Ultrafiltration and Ultracentrifugation Studies

Ultrafiltration experiments are highly suitable to examine the changes in the albumin-bound fraction of ligands, because HSA (66.5 kDa) and HSA-bound molecules cannot pass through the filter unit with 30 kDa MWCO value [35,50,51]. Based on this principle, the displacement of the ligand from HSA results in its elevated concentration [49], while the increased binding affinity of the ligand toward the protein leads to its lower concentration in the filtrate [52]. Importantly, in ultrafiltration experiments, we directly analyze the concentration of the Site I marker WAR with HPLC-FLD in the HSA-free filtrate. Since we can avoid the unexpected spectroscopic effects resulting from the cooperative binding of the two ligand molecules to albumin, these studies provide more reliable data than the typical spectroscopic investigations. Ultrafiltration studies were also performed without HSA, where the presence of Heme site markers did not affect the filtered fraction of WAR.

**Figure 6:**
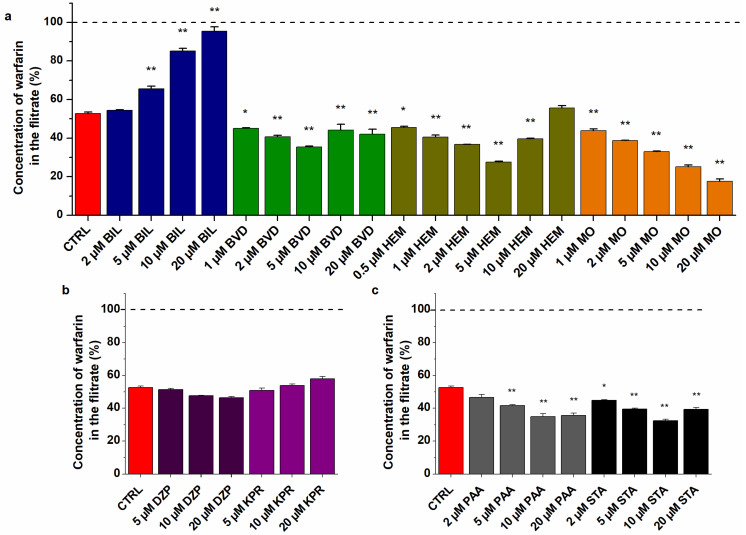
Effects of Heme site ligands (**a**) Sudlow’s site II markers (**b**) and fatty acids (**c**) on the filtered fraction of WAR. Before ultrafiltration, samples contained WAR (1.0 μM) and HSA (5.0 μM) in the presence of palmitic acid (PAA), stearic acid (STA), diazepam (DZP), ketoprofen (KPR), BIL, BVD, HEM, or MO (0–20 μM) in PBS (pH 7.4; * *p* < 0.05, ** *p* < 0.01). The filtered concentration of WAR was compared to its filtered concentration measured in the absence of HSA (100%, see with dashed line). WAR was quantified in the filtrates with HPLC-FLD (see details in Section 3.6).

Ultrafiltration studies demonstrated the distinct and very complex impacts of Heme site ligands on the albumin binding of WAR (Figure 6a). In a concentration-dependent fashion, BIL strongly increased the amount of WAR in the filtrate. Thus, albumin binding of WAR was disrupted (5 μM) then almost completely abolished (20 μM) by BIL (Figure 6a). In contrast, MO significantly reduced the filtered fraction of WAR even at 1 μM concentrations, after which a further gradual decrease was observed in the presence of higher amounts of MO (2–20 μM) (Figure 6a). These data demonstrate that MO can strongly increase the binding affinity of WAR toward the protein due to their allosteric interaction. Furthermore, lower concentrations of HEM (1–5 μM) decreased the filtered fraction of WAR; however, in the presence of 10 and 20 μM HEM, this effect was reversed and a gradual elevation was observed (Figure 6a). BVD induced a similar impact to HEM, but it was less pronounced. Based on the complex impacts of HEM and BVD, it is reasonable to hypothesize that these ligands have secondary binding sites on HSA. The lower or comparable concentrations of BVD or HEM with HSA (5 μM used in this model) led to the interaction with their high-affinity binding site (Heme site), resulting in the positive allosteric modulation of WAR–HSA interaction. However, the considerably higher concentrations of BVD and HEM (2- or 4-fold) compared to HSA may cause their interaction with a secondary binding site on the protein, which can directly or indirectly interfere with WAR–HSA interaction. A previous study also suggested that HEM has lower-affinity binding sites on albumin [25], while we did not find data in regard to a secondary binding site of BVD. Nevertheless, the lower molar concentrations of BVD and HEM showed similar impact to MO: they enhanced the interaction of WAR with the protein (Figure 6). 

In agreement with fluorescence spectroscopic studies (Figure 5f), Site II ligands did not modify significantly the concentration of WAR in the filtrate (Figure 6b), only a very slight decrease and increase were caused by diazepam and ketoprofen, respectively. Some studies suggest the allosteric interactions of certain Site II and Site I ligands [53,54]; however, based on our results, it does not seem relevant.

Palmitic acid and stearic acid decreased the filtered fraction of WAR (Figure 6c), suggesting that they can increase the binding affinity of the Site I ligand toward HSA. This observation is in agreement with earlier studies [7,55]. Furthermore, our data demonstrated that the fatty-acid-induced strong elevation in the fluorescence of WAR (Figure 5f) was caused by two components: (1) fatty acids increase the bound fraction of WAR, which consequently elevates the emission signal of the ligand and (2) fatty acids increase the fluorescence signal of the WAR–HSA complex during their cooperative binding to the protein.

**Figure 7:**
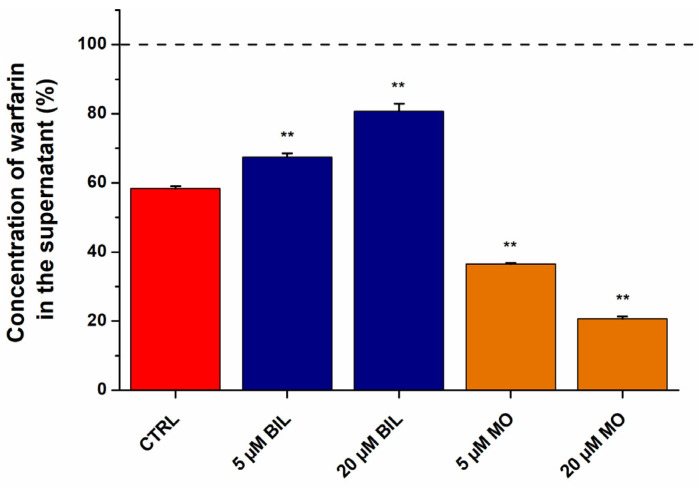
Concentration of WAR in the supernatant after ultracentrifugation (170,000× *g*, 16 h, 20 °C) of the protein fraction (** *p* < 0.01). Samples contained WAR (1.0 μM) and HSA (3.0 μM) in the presence of BIL or MO (0, 5, or 20 μM) in PBS (pH 7.4). The concentration of WAR in the supernatant was compared to its concentration measured without HSA (100%, see with dashed line). WAR was quantified in the supernatants with HPLC-FLD (see details in Section 3.6).

In order to confirm the results of ultrafiltration studies, ultracentrifugation experiments were also performed. In the latter model, the protein fraction was centrifuged (with the bound ligands) [13,56,57], after which the unbound free fraction of WAR was directly quantified in the supernatant by HPLC-FLD. Since BIL and MO showed opposite effects in ultrafiltration studies and they produced the largest changes in the filtered fraction of WAR (Figure 6a), we tested their impacts on WAR–HSA interaction in ultracentrifugation experiments. BIL significantly increased while MO considerably decreased the unbound fraction of WAR in the supernatant (Figure 7), which confirms the results of ultrafiltration experiments (Figure 6).

In our previous experiments, when we examined the competitive interactions of Site I ligands with WAR, the results of fluorescence spectroscopic (changes in the fluorescence of WAR) and ultrafiltration models were in good agreement [49,50,51]. If two ligands compete for the same binding site, then these ligands are not able to simultaneously occupy it. Consequently, the decreased fluorescence emission signal of WAR is clearly resulted from its displacement from HSA. However, the cooperative binding of two ligand molecules to different sites of albumin can cause changes not only in the binding affinity, but the fluorescence signal of the formed complexes may also be affected. The latter phenomenon explains why we obtained misleading results from fluorescence spectroscopic experiments in the diligand studies (Figure 5). In the presence of Heme site markers, the emission signal of WAR considerably decreased, while ultrafiltration experiments demonstrated that only BIL caused the strong displacement of WAR (Figure 6). Interestingly, among the Heme site ligands tested, BIL induced the smallest reduction in the emission intensity of WAR in fluorescence spectroscopic studies (Figure 5). BVD, HEM, and MO also strongly decreased the fluorescence signal of the Site I marker (Figure 5); in contrast, ultrafiltration studies demonstrated that these Heme site ligands can increase the bound fraction of WAR (Figure 6). Thus, the cooperative binding of BVD, HEM, or MO considerably reduces the emission signal of the WAR–HSA complex. These observations strongly underline that the results of fluorescence spectroscopic studies should be carefully evaluated in diligand models and the application of other confirmatory techniques is indispensable to avoid false conclusions.

## 3. Materials and Methods

### 3.1. Reagents

Racemic warfarin, bilirubin, biliverdin, hemin, and human serum albumin (Product No.: A1653) were purchased from Merck (Darmstadt, Germany). Methyl orange was from Reanal (Budapest, Hungary), while HPLC-grade acetonitrile and methanol were obtained from VWR (Budapest, Hungary). To mimic extracellular physiological conditions, measurements were performed in phosphate-buffered saline (PBS, pH 7.4).

### 3.2. UV-Vis Spectroscopic Studies

UV-Vis spectra were recorded in PBS (pH 7.4) at 25 °C, employing a Jasco V730 UV-Vis spectrophotometer (Tokyo, Japan). Absorption spectra of Heme site ligands (5.0 μM each) have been recorded without and with increasing concentrations of HSA (0.0, 0.2, 0.5, 0.7, 1.0, 1.5, 2.0, 5.0, 10, and 15 μM). Binding constants (*K*) of ligand–albumin complexes were determined based on the HSA-induced increase in their absorbance (at 480 nm for BIL, at 390 nm for BVD, at 400 nm for HEM, and at 460 nm for MO). Under the applied conditions, HSA did not show absorbance at the wavelengths used for evaluation. Binding constants (*K*; unit: L/mol) were calculated with linear and non-linear fitting, using the Scatchard equation [58] and the Hyperquad2006 software [59], respectively. The Scatchard equation for 1:1 binding model follows:

(1) $$\frac{\left(A-A_{0}\right)}{{\left[HSA\right]}_{0}}=\left(A_{ligand-HSA}-A_{0}\right)\times K-\left(A-A_{0}\right)\times K$$

where *A*_0_ and *A* are the absorbance values of the ligand in the absence and presence of HSA, respectively; *A_ligand-HSA_* is the absorbance when each ligand molecule bound to HSA, and *[HSA]* is the concentration of albumin (unit: mol/L). 

The following equations are implemented in the Hyperquad program code:

(2) $$aLIGAND+bHSA\;↔LIGAND_{a}HSA_{b}$$

(3) $$\beta_{ab}=\frac{\left[LIGAND_{a}HSA_{b}\right]}{{\left[LIGAND\right]}^{a}{\left[HSA\right]}^{b}}$$

where *a* and *b* indicate the stoichiometry associated with the equilibria in the solution. All equilibrium constants are defined as overall association constants (*β*):

(4) $$LIGAND+HSA\;↔LIGAND\;HSA;\;\beta_{1}=\frac{\left[LIGAND\;HSA\right]}{\left[LIGAND\right]\left[HSA\right]}$$

(5) $$2\;LIGAND+HSA\;↔LIGAND_{2}\;HSA;\;\beta_{2}=\frac{\left[LIGAND_{2}HSA\right]}{{\left[LIGAND\right]}^{2}\left[HSA\right]}$$

(6) $$aLIGAND+HSA\;↔LIGAND_{a}HSA;\;\beta_{a}=\frac{\left[LIGAND_{a}HSA\right]}{{\left[LIGAND\right]}^{a}\left[HSA\right]}$$

The stepwise association constants (*K*) are defined by *β*:

(7) $$\beta_{1}=K_{1};\;\beta_{2}=K_{1}\times K_{2};\;\beta_{a}=K_{1}\times K_{2}\times \ldots \times K_{a}$$

During data evaluation the stoichiometry and association constants of the ligand binding process were calculated using the lowest standard deviation model.

### 3.3. Fluorescence Spectroscopic Studies

Fluorescence spectra were recorded in PBS (pH 7.4) at 25 °C, employing a Hitachi F-4500 fluorometer (Tokyo, Japan). Binding constants of BIL, BVD, HEM, and MO were also determined based on fluorescence quenching studies (λ_ex_ = 295 nm). Emission spectra of HSA (2.0 μM) were recorded in the absence and presence of the increasing concentrations of Heme site ligands (0.0, 0.5, 1.0, 2.0, 3.0, 5.0, 7.0, 10, and 15 μM). Before the evaluation of emission intensity data at 340 nm, the inner-filter effect of ligand molecules was corrected using the following equation [60,61]:

(8) $$I_{cor}=I_{obs}\times e^{\left(A_{ex}+A_{em}\right)/2}$$

where *I_cor_* and *I_obs_* are the corrected and observed fluorescence emission intensities, respectively; *A_ex_* and *A_em_* are the absorbance values of Heme site ligands at 295 and 340 nm, respectively.

Ligand–HSA interactions were evaluated with linear and non-linear fitting, using the graphical application of the Stern–Volmer equation [60] and the Hyperquad software [59], respectively. Stern–Volmer equation has been described as:

(9) $$\frac{I_{0}}{I}=1+K_{SV}\times \left[Q\right]$$

where *I*_0_ and *I* are the fluorescence intensities of HSA in the absence and presence of Heme site ligands, respectively; *K_SV_* is the Stern–Volmer quenching constant (unit: L/mol) and [*Q*] is the molar concentration of the quencher (Heme site ligands). The Hyperquad program code used is defined in Equations (2)–(7).

In another experiment, fluorescence quenching studies were also performed in the presence of standard concentrations of HSA (2.0 μM) and Heme site ligands (each 1.0 μM), with increasing concentrations of the Site I marker WAR (0.0, 0.3, 0.6, 1.0, 1.5, 2.0, 2.5, 3.0, 3.5, and 4.0 μM). Thereafter, the changes in the fluorescence emission signal at 340 nm were compared and evaluated (λ_ex_ = 295 nm).

Finally, the effects of BIL, BVD, HEM, and MO were tested on the fluorescence signal of WAR–HSA complex. The interaction of WAR with HSA results in the strong increase in its fluorescence emission signal of WAR at 379 nm (λ_ex_ = 317 nm) [12]. Therefore, the displacement of WAR from albumin leads to the considerable decrease in its emission intensity [12,50], while the elevated fluorescence may suggest the increased binding affinity of WAR toward HSA [52]. Emission spectra of WAR (1.0 μM) were recorded in the presence of HSA (3.5 μM) and increasing concentrations of Heme site ligands (0.0, 0.5, 1.0, 2.5, 5.0, and 10 μM).

### 3.4. Ultrafiltration Studies

Effects of BIL, BVD, HEM, and MO on the albumin-bound fraction of the Site I ligand WAR were examined by ultrafiltration [35,49]. Amicon Ultra-0.5 centrifugal filters (Merck, Darmstadt, Germany) with 30 kDa molecular weight cut-off (MWCO) value were applied. Since HSA (66.5 kDa) and albumin-bound molecules cannot pass through the filter, the changes in the bound fraction of WAR strongly affect its concentration in the filtrate [49,62]. Before ultrafiltration, filter units were washed once with water (500 μL) then once with PBS (500 μL). Samples contained WAR and HSA (1.0 μM and 5.0 μM, respectively) in the absence and presence of Heme site ligands (0–20 μM) in PBS (pH 7.4). A 500 μL aliquot of samples was transferred to the filter and centrifuged for 10 min at 7500 g and 25 °C (fixed angle rotor). Thereafter, WAR concentrations in the filtrate were directly analyzed by HPLC-FLD (see in Section 3.6). 

### 3.5. Ultracentrifugation Studies

With the optimal conditions of ultracentrifugation, we can sediment the albumin fraction of a solution without the disruption of ligand–HSA interactions [13,56,57]. Consequently, the free fraction of a ligand can be quantified from the supernatant. To confirm the results of ultrafiltration studies, the effects of BIL and MO on the free fraction of WAR were also examined with ultracentrifugation. Samples contained WAR and HSA (1.0 μM and 3.0 μM, respectively) in PBS (pH 7.4) without and with BIL or MO (5.0 μM or 20 μM). In 11 × 34 mm PC tubes (Beckman Coulter, Brea, CA, USA), a 900 μL aliquot of samples was centrifuged for 16 h at 170,000× *g* and 20 °C applying a Beckman Coulter Optima MAX-XP tabletop ultracentrifuge (with MLA-130 fixed-angle rotor) [13]. Then a 200 μL volume of the supernatant was carefully removed and directly analyzed by HPLC-FLD (see in Section 3.6).

### 3.6. HPLC Analyses

We applied an integrated HPLC system (Jasco, Tokyo, Japan) built up from an autosampler (AS-4050), a binary pump (PU-4180), and a fluorescence detector (FP-920). Chromatographic data were evaluated employing ChromNAV2 software (Jasco, Tokyo, Japan). WAR concentrations were quantified using the previously reported method [35,50]. Briefly, samples (20 μL) were driven through a guard column (SecurityGuard C18, 4.0 × 3.0 mm; Phenomenex, Torrance, CA, USA) linked to a Nova-Pak C18 analytical column (150 × 3.9 mm, 4 μm; Waters, Milford, MA, USA). The isocratic elution was performed at room temperature with 1.0 mL/min flow rate applying sodium phosphate buffer (20 mM, pH 7.0), methanol, and acetonitrile (70:25:5 *v*/*v*%) as the mobile phase. WAR was detected at 390 nm (λ_ex_ = 310 nm).

### 3.7. Statistics

Statistical significance was established by one-way ANOVA (with Tukey’s post hoc) test, employing SPSS Statistics software (IBM, Armonk, NY, USA). The level of significance was set to *p* < 0.05 and *p* < 0.01.

## 4. Conclusions

The interactions of certain Heme site ligands (BIL, BVD, HEM, and MO) with HSA were tested employing UV-Vis and fluorescence quenching studies, where data were evaluated with both linear and non-linear fittings. These investigations underline that different spectroscopic techniques and data evaluation strategies can strongly affect the binding constants determined. Furthermore, the impacts of the above-listed Heme site ligands were examined on the interaction of the Site I ligand WAR with HSA, employing fluorescence spectroscopic, ultrafiltration, and ultracentrifugation techniques. Quenching studies gave only semi-quantitative results and showed no relevant effects of Heme site ligands on WAR–HSA interaction. The other fluorescent model (which examined the changes in the fluorescence signal of WAR) suggested that each Heme site ligand strongly decreased the albumin binding of WAR. However, ultrafiltration and ultracentrifugation studies demonstrated the complex modulation of WAR–HSA interaction by the different Heme site markers. In a concentration-dependent fashion, BIL strongly decreased while MO considerably increased the bound fraction of warfarin. Moreover, the low concentrations of BVD and HEM enhanced the albumin binding of WAR, while their higher concentrations caused a gradual increase in the filtered fraction of the Site I ligand. Another interesting observation is the fatty acid-induced remarkable increase in the emission signal of WAR, due to the increased albumin binding of the Site I ligand and the elevated fluorescence of the WAR–HSA complex in the presence of palmitic acid and stearic acid. Herein, we present novel data in regard to the impacts of BIL, BVD, HEM, and MO on the albumin binding of the Site I marker drug WAR. Our study highlights the complex modulation of WAR–HSA interaction by different Heme site ligands and strongly underlines the limitations of fluorescence spectroscopic studies in diligand–albumin models.

## Figures and Tables

**Table 1:** Decimal logarithmic values of the binding constants (*K*; unit: L/mol) and the Stern–Volmer quenching constants (*K_SV_*; unit: L/mol) of BIL–HSA, BVD–HSA, HEM–HSA, and MO–HSA complexes based on UV-Vis and fluorescence spectroscopic studies. Mean ± SEM values represented are at least from three independent experiments. Data were evaluated applying both linear (Scatchard plot, Equation (1); Stern–Volmer plot, Equation (9)) and non-linear (Hyperquad, Equations (2)–(7)) fitting. Since the Stern–Volmer plot of HEM–HSA complex showed two linear sections, we also evaluated these data in the 0.5–3 μM and in the 3–15 μM concentration ranges.

	UV-Vis Spectroscopy		Fluorescence Quenching	
	*Scatchard Plot* log*K*	*Hyperquad* log*K*	*Stern–Volmer Plot* log*K_SV_*	*Hyperquad* log*K*
**BIL**	5.83 ± 0.02	7.53 ± 0.02	5.26 ± 0.01	5.35 ± 0.01
**BVD**	4.96 ± 0.06	5.55 ± 0.11	5.37 ± 0.03	5.45 ± 0.02
**HEM**	5.74 ± 0.04	7.35 ± 0.03	5.54 ± 0.02 *0.5–3* µM*: 5.23 ± 0.02* *3–15* µM*: 5.55 ± 0.03*	5.49 ± 0.02 *0.5–3* µM*: 5.34 ± 0.01* *3–15* µM*: 5.53 ± 0.02*
**MO**	4.19 ± 0.09	4.74 ± 0.08	4.76 ± 0.02	4.83 ± 0.02

## Data Availability

Data will be made available on request.

## Supplementary Materials

The following supporting information can be downloaded at: https://www.mdpi.com/article/10.3390/ijms232214007/s1.
