# Supplementary material for: Effects of Heme Site (FA1) Ligands Bilirubin, Biliverdin, Hemin, and Methyl Orange on the Albumin Binding of Site I Marker Warfarin: Complex Allosteric Interactions
Source: Int J Mol Sci. 2022 Nov 13;23(22):14007. doi: 10.3390/ijms232214007 (PMC9694159; doi:10.3390/ijms232214007)
Supplement: Supplementary file 1 [file ijms-23-14007-s001.zip › ijms-1985695-supplementary.pdf]

Effects of Heme Site (FA1) Ligands Bilirubin, Biliverdin, Hemin, and Methyl Orange on the Albumin Binding of Site I Marker Warfarin: Complex Allosteric Interactions

SUPPLEMENTARY MATERIAL

Beáta Lemli,[1,2] Zuzana Lomozová,[3] Tamás Huber,[4] András Lukács,[4] Miklós Poór [1,5,*]

[1] Department of Pharmacology, Faculty of Pharmacy, University of Pécs, Rókus u. 2, H-7624 Pécs, Hungary

[2] Green Chemistry Research Group, János Szentágothai Research Center, University of Pécs, Ifjúság útja 20, H-7624 Pécs, Hungary

[3] The Department of Pharmacognosy and Pharmaceutical Botany, Faculty of Pharmacy in Hradec Králové, Charles University, Akademika Heyrovského 1203, 500 05 Hradec Králové, Czech Republic

[4] Department of Biophysics, Medical School, University of Pécs, Szigeti út 12, H-7624 Pécs, Hungary

[5] Lab-on-a-Chip Research Group, János Szentágothai Research Centre, University of Pécs, Ifjúság útja 20, H-7624 Pécs, Hungary

* Correspondence: poor.miklos@pte.hu; Tel: +36-72-501-500 ext: 28316

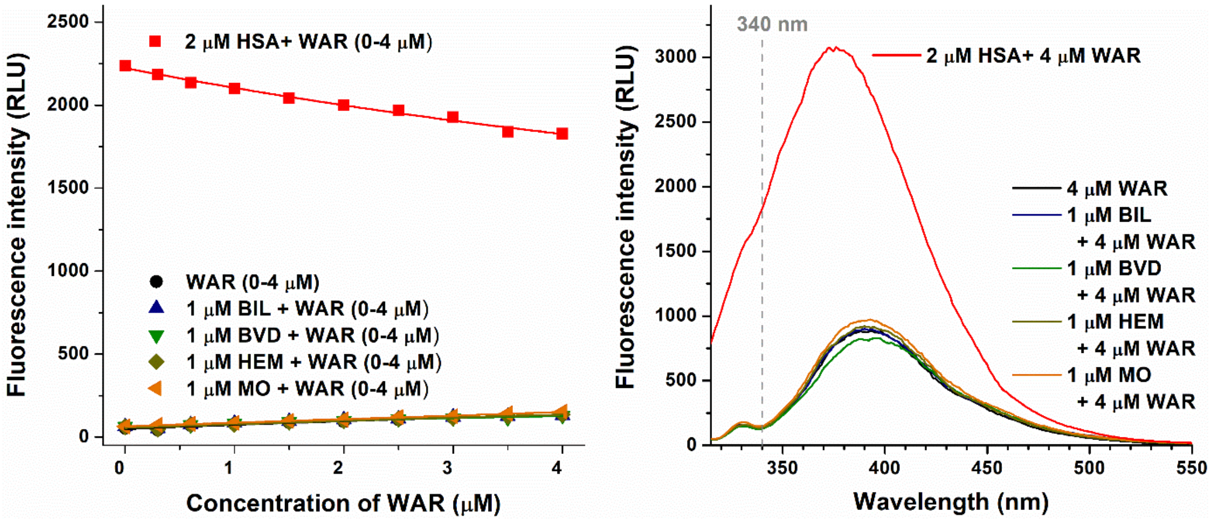

Figure S1: Fluorescence emission signal (at 340 nm) and representative emission spectra of warfarin in the absence and presence of Heme site ligands. ***Left panel***: Fluorescence emission signal of albumin (HSA, 2 μM) in the presence of increasing concentrations warfarin (WAR, 0-4 μM) (**red**). Fluorescence emission signal of WAR (0-4 μM) without (**black**) and with bilirubin (BIL, 1 μM; **blue**), biliverdin (BVD, 1 μM; **green**), hemin (HEM, 1 μM; **dark yellow**), and methyl orange (MO, 1 μM; **orange**) in PBS (pH 7.4; λ_{ex} = 295 nm, λ_{em} = 340 nm). ***Right panel***: Fluorescence emission spectrum of HSA in the presence of WAR (**red**). Fluorescence emission spectrum of WAR without (**black**) and with BIL (**blue**), BVD (**green**), HEM (**dark yellow**), and MO (**orange**) in PBS (pH 7.4; λ_{ex} = 295 nm).

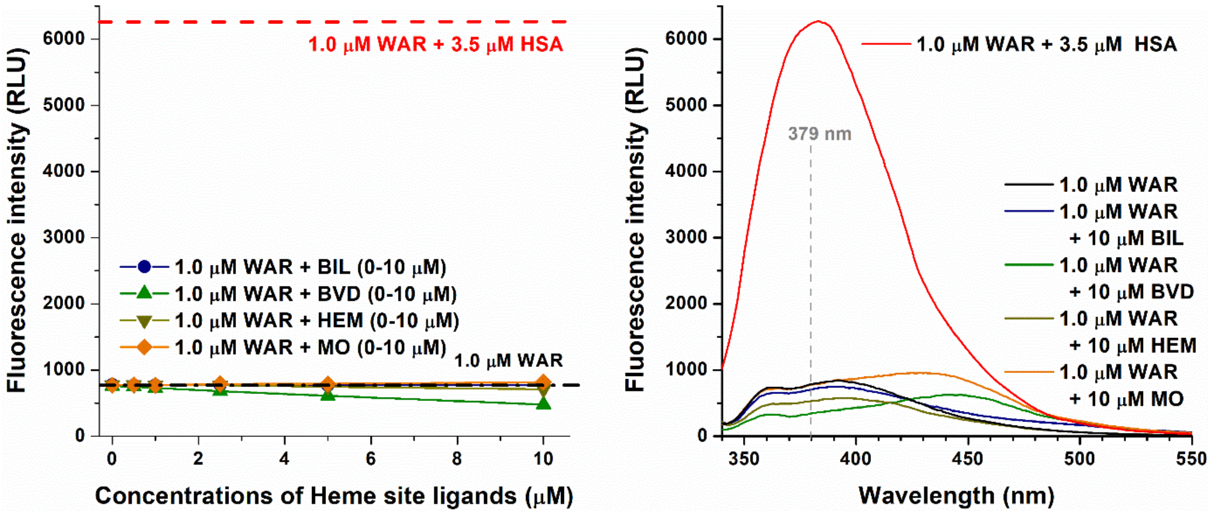

Figure S2: Fluorescence emission signal (at 379 nm) and representative emission spectra of warfarin in the absence and presence of Heme site ligands. ***Left panel***: Fluorescence emission signal of warfarin-HSA complex (**red dashed line**), warfarin (WAR) alone (**black dash-dotted line**), and WAR in the presence of increasing concentrations of bilirubin (BIL; **blue**), biliverdin (BVD; **green**), hemin (HEM; **dark yellow**) and methyl orange (MO; **orange**) in PBS (pH 7.4; λ_{ex} = 317 nm, λ_{em} = 379 nm). ***Right panel***: Fluorescence emission spectra of WAR-HSA complex (**red**), WAR alone (**black**), and WAR in the presence of BIL (**blue**), BVD (**green**), HEM (**dark yellow**), and MO (**orange**) in PBS (pH 7.4; λ_{ex} = 317 nm).

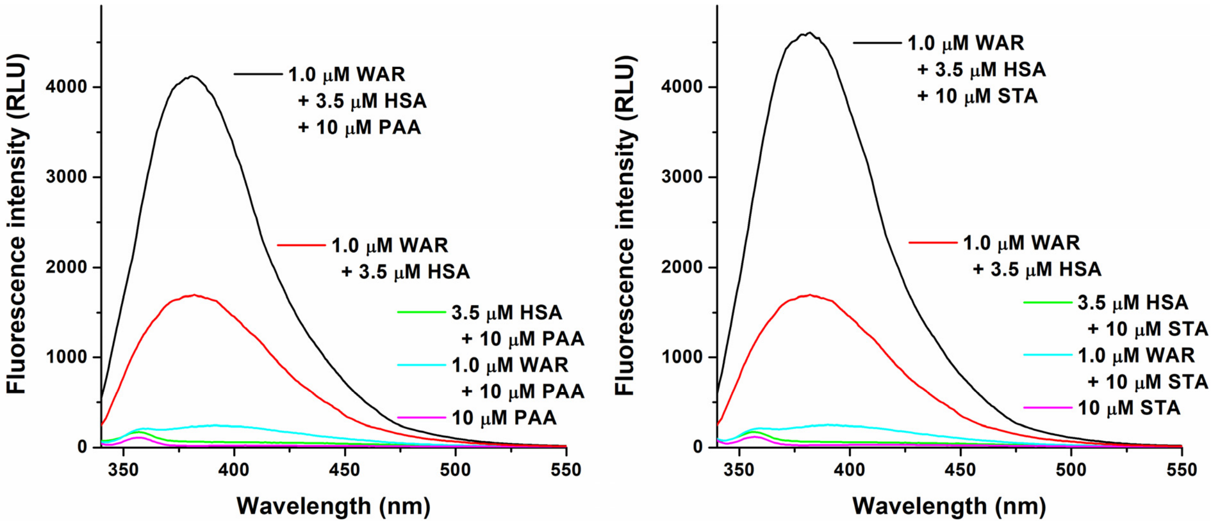

Figure S3: Representative emission spectra of palmitic acid and stearic acid without and with warfarin, albumin, and warfarin + albumin. Emission spectra of palmitic acid (PAA, 10 μM; ***left panel***) and stearic acid (STA, 10 μM; ***right panel***) in the absence and presence of warfarin (WAR, 1.0 μM), albumin (HSA, 3.5 μM), and WAR-HSA complex in PBS (pH 7.4; λ_{ex} = 317 nm).
